# Molecular Mechanisms of Cachexia: A Review

**DOI:** 10.3390/cells13030252

**Published:** 2024-01-29

**Authors:** Mahdi Neshan, Diamantis I. Tsilimigras, Xu Han, Hua Zhu, Timothy M. Pawlik

**Affiliations:** 1 Department of General Surgery, Shahid Sadoughi University of Medical Sciences and Health Services, Yazd 8915887857, Iran; mahdineshan.research@gmail.com; 2 Department of Surgery, The Ohio State University Wexner Medical Center and James Comprehensive Cancer Center, Columbus, OH 43210, USA; diamantis.tsilimigras@osumc.edu (D.I.T.); xu.han@osumc.edu (X.H.); hua.zhu@osumc.edu (H.Z.)

**Keywords:** cancer cachexia, muscular wasting, atrophy, weight loss, cancer-related syndrome, skeletal muscle

## Abstract

Cachexia is a condition characterized by substantial loss of body weight resulting from the depletion of skeletal muscle and adipose tissue. A considerable fraction of patients with advanced cancer, particularly those who have been diagnosed with pancreatic or gastric cancer, lung cancer, prostate cancer, colon cancer, breast cancer, or leukemias, are impacted by this condition. This syndrome manifests at all stages of cancer and is associated with an unfavorable prognosis. It heightens the susceptibility to surgical complications, chemotherapy toxicity, functional impairments, breathing difficulties, and fatigue. The early detection of patients with cancer cachexia has the potential to enhance both their quality of life and overall survival rates. Regarding this matter, blood biomarkers, although helpful, possess certain limitations and do not exhibit universal application. Additionally, the available treatment options for cachexia are currently limited, and there is a lack of comprehensive understanding of the underlying molecular pathways associated with this condition. Thus, this review aims to provide an overview of molecular mechanisms associated with cachexia and potential therapeutic targets for the development of effective treatments for this devastating condition.

## 1. Introduction

Cachexia is a pathological state characterized by substantial loss of body weight resulting from the depletion of skeletal muscle and body fat, which cannot be effectively counteracted by conventional nutritional interventions. Cancer cachexia is characterized by the presence of inflammation and a decrease in body weight in the setting of cancer. The diagnostic criteria for cancer cachexia include a weight reduction of 5% within a six-month period or a reduction of 2% among individuals with sarcopenia [1,2,3,4,5]. Cachexia is a complex metabolic condition that affects a substantial proportion of individuals with advanced cancer. The prevalence of the syndrome varies depending on the specific tumor type. For instance, pancreatic or gastric cancer patients have a higher incidence, with over 80% experiencing cancer cachexia. In comparison, roughly 50% of patients with lung, prostate, or colon cancer are affected by cancer cachexia, while around 40% of individuals with breast tumors or certain leukemias experience the condition [6].

Cachexia is a paraneoplastic syndrome that can occur during any stage of cancer and is generally associated with a poor prognosis. Specifically, cancer cachexia has been associated with a higher risk of surgical complications, chemotherapy toxicity, functional limitations, respiratory challenges, and fatigue—all of which can have detrimental effects on patient quality of life and overall survival [1,7]. Cachexia has been described as an “auto-cannibalism condition”, which occurs when tumors utilize the host’s resources to support their own growth. The metabolic alterations observed in cachexia are commonly attributed to immunological and neuroendocrine reactions, like the physiological characteristics of trauma or sepsis. Cancer cachexia may be attributed to variations in genetic events or gene expression [8,9]. The early detection of cancer patients with cachexia can potentially lead to interventions to counteract this process and improve quality of life and overall survival. Several blood biomarkers have been proposed to identify cancer cachexia; however, their clinical utility has been limited only to certain cancer types and they have not been universally adopted in the clinical setting [10,11,12].

The molecular mechanisms responsible for the development of cancer cachexia have not been fully defined [13,14]. We, herein, provide a review of the pathophysiology of cachexia, as well as highlight the biomarkers and characterize the molecular mechanisms underlying the development of cancer cachexia in light of potential future therapeutic strategies.

## 2. Pathophysiology

**Figure 1:**
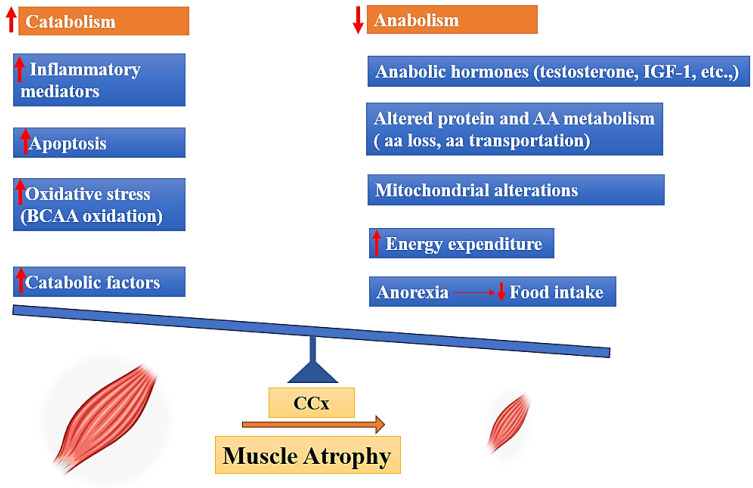
Drivers of muscle mass atrophy in cancer cachexia, BCAA: (Branched-chain amino acids), IGF-1: (Insulin-like Growth Factor 1), AA: (amino acid).

Cancer cachexia is a pathological condition characterized by an imbalance in the equilibrium between anabolic and catabolic processes. Skeletal muscle, which serves as a substantial reservoir of proteins, is regulated by catabolic processes that facilitate the breakdown of proteins or hinder their synthesis, as well as anabolic mechanisms that enhance protein synthesis [15]. Several factors can disturb the equilibrium of skeletal muscle homeostasis. These factors include a decrease in anabolic hormone levels, loss of amino acids, insufficient food intake, and muscle inactivity; in contrast, other factors can enhance catabolism, such as elevated cytokine levels and oxidative stress (Figure 1) [4,16,17,18,19]. 

In this context, muscle can demonstrate a decreased sensitivity to anabolic hormones, including growth hormone, insulin, IGF-1, glucocorticoids, and testosterone. This decrease is associated with the suppression of transcription and expression of genes associated with anabolic or catabolic processes [16]. Impaired protein turnover is a common characteristic of the excessive energy loss observed in cancer cachexia, both in patients and in experimental animals. Indeed, muscle proteolysis is activated along with a state of hypo-anabolism that alters mitochondrial function [20]. In the context of cancer cachexia, amino acids (AAs) are captured by the tumor and other metabolically active tissues including the liver. These AAs are sourced from lean tissues, particularly the skeletal muscle. This process is facilitated by the activation of two primary proteolytic systems, namely the proteasome and the autophagic-lysosomal pathway. The activation of these systems is triggered by inflammation-dependent transcription factors, such as Forkhead box protein O1/3 (FoxO1/3) and nuclear factor-κB (NF-κB) [21]. Previous studies demonstrated a hyper-activation of the autophagic system, inflammation, and increased reactive oxygen species (ROS) in the muscle of tumor-bearing animals as well as in cancer patients [22,23]. Pro-oxidant species damage the mitochondria, leading to further ROS production and stimulating mitophagy, affecting mitochondrial abundance in the muscle. Since mitochondria represent the main producers of energy required for contraction, alterations of their homeostasis impair muscle function [22,23]. In parallel, antioxidant enzymes, such as superoxide dismutase (SOD), catalase, and glutathione peroxidase (GPx), are up-regulated in muscle of tumor-bearing animals in an attempt to counteract the oxidative insult, albeit not enough to maintain the redox balance, while others have reported a down-regulation of the same enzymes, further promoting oxidative stress [24]. Cancer cachexia involves the degradation of muscle protein, which provides AAs required for the creation of inflammatory proteins [23]. Several reports have noted a correlation between inflammation and cachexia, with inflammation being implicated as an important factor in weight loss among cancer patients [3,25]. Inflammatory cytokines including tumor necrosis factor-alpha (TNF-α) and interleukin-6 (IL-6) induce the activation of the transcription factor nuclear factor κ-light-chain enhancer of activated B cells (NF-κB), which results in the inhibition of MyoD synthesis, a muscle-specific transcription factor that plays a crucial role in muscle differentiation [26]. Cytokines are also involved in the regulation of anorexigenic and orexigenic pathways inside the hypothalamus, leading to the development of anorexia which might also contribute to the development of cachexia [27]. In addition, increased concentrations of possible catabolic mediators, such as cortisol, glucagon, and adrenaline, as well as oxidative stress, can contribute to the pathophysiology of cachexia [16].

## 3. Biomarkers and Prognostic Role

Several biomarkers may correlate with cachexia, including cachexia-inducing factors [9,28], pro-inflammatory cytokines [29,30], lipids [31], protein and fat product degradation [32], and microRNAs (Table 1) [33,34]. Given that cachexia is characterized by significant muscle mass loss, previous research has largely focused on identifying biomarkers associated with muscle wasting. 

In particular, the association of cytokine levels, weight reduction, and lean mass among patients with lung, gastrointestinal, and pancreatic malignancies has been investigated. For example, Lerner et al. noted an inverse correlation between circulating levels of growth/differentiation factor 15 (GDF-15) and activin A (ActA) and lean mass, suggesting that these biomarkers may be promising lead indicators of weight loss and cachexia [35,36]. A number of host and tumor-related biomarkers have been correlated with cachexia in various types of cancers [34,37]. Of note, none of these biomarkers are currently approved as standard of care diagnostic tools to detect cancer cachexia in clinical practice.

## 4. Molecular Mechanism and Mediators

The molecular mechanisms implicated in the process of cachexia have been the focus of research as a means to develop innovative therapeutic approaches to halt tumor-induced depletion of adipose tissue and skeletal muscle [38,39]. Several mediators that contribute to cancer cachexia originate from targeted mesenchymal tissues, as well as immunological or tumor cells. 

### 4.1. Muscle Atrophy 

Cachexia involves an imbalance between muscle protein synthesis and degradation. Several mediators that contribute to this phenomenon include transforming growth factor-beta (TGF-β), TNF-α, interleukins, etc. In particular, the TGF-β superfamily of proteins plays an important role in cancer cachexia [9]. With the TGF-β superfamily, proteins, such as activins A and B, as well as myostatin (MSTN), have been investigated the most in the context of cachexia. These proteins are potent inhibitors of muscle growth, and multiple preclinical studies have demonstrated elevated levels of these factors in cachectic animal models [40,41].

**Figure 2:**
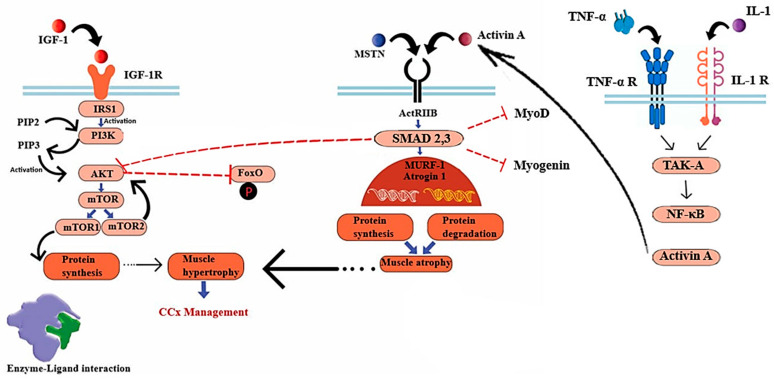
Cachexia-related pathways in skeletal muscle: The mTOR (mechanistic Target of Rapamycin) signaling pathway, which is initiated by activated Akt and inhibits the phosphorylation of FoxO, facilitates the process of protein synthesis. The pathways involved in atrophy encompass the myostatin/Activin ligands, which exert their effects through the ACTRII receptor and SMAD 2,3. These ligands impede protein synthesis mediated by Akt and inhibit MyoD and myogenin (genes associated with muscle differentiation). SMAD 2,3 signaling can also become activated by Activin A which is induced by IL-1 and TNF- α). Abbreviations: PI3K (phosphatidylinositol-3-kinase), PIP_2_ (phosphoinositide-4, 5-biphosphate), PIP_3_ (phosphoinositide-3,4,5-triphosphate).

In addition, the inhibition of these members of the TGF-β superfamily can effectively mitigate muscle atrophy in animal cancer models [42,43]. Cachexia also involves an interplay between signaling pathways such as insulin-like growth factor 1 (IGF-1) and MSTN (Figure 2) [9,44,45]. IGF-1 is a hormone that plays a crucial role in promoting muscle growth by regulating both anabolic and catabolic pathways [46]. The formation of insulin receptor substrate binding sites (IRSs) occurs upon the binding of IGF-1 to its receptor, leading to the activation and subsequent auto-phosphorylation of the receptor intrinsic tyrosine kinase [47].

Phosphorylated IRSs lead to protein synthesis through activating Akt [45], which in turn, triggers the activation of the mammalian target of rapamycin complexes 1,2 (mTORC1,2) [48]. In the context of cellular development, mTORC1 plays a crucial role in regulating the activity of unc-51-like autophagy-activating kinase 1 (ULK1) and ATG13. This regulation leads to a reduction in catabolic autophagy and an enhancement in protein synthesis mediated by 4E-BPs and p70S6K [49,50]. In another related pathway, MSTN makes its negative impact on muscle mass regulation by inducing ActRIIB, which phosphorylates SMAD2 and SMAD3, inhibiting Akt protein [51,52]. Alterations in these signaling pathways transcriptionally activate two key muscle atrophy factors, muscle RING-finger protein-1 (MURF-1) and muscle atrophy F-box (MAFbx, also known as Atrogin-1) [53]. Atrogin-1 and MuRF-1 (Trim63) are E3 ubiquitin ligases that are prominently expressed in skeletal and cardiac muscle tissues and perform critical functions related to muscle remodeling. The transcription of the Atrogin-1 and MuRF1 genes is increased in skeletal muscle atrophy, while a decrease in protein expression has been associated with cardiac hypertrophy [54,55]. The promotion of muscle growth is facilitated by the IGF-1/PI3K/AKT axis, which acts through the activation of the mTOR kinase. Additionally, this axis inhibits the expression of MURF1 and Atrogin-1 by suppressing the activity of the FOXO transcription factor. FOXO is responsible for regulating the expression of atrogenes involved in protein degradation [56]. The primary role of FOXO transcription factors is to facilitate the regulation of E3 ubiquitin ligases in the context of muscle atrophy. Myotubes experiencing atrophy exhibit the activation of FOXO transcription factor and an upregulation of Atrogin-1 gene expression. Therefore, the continuous activation of FOXO3 leads to an upregulation of Atrogin-1 expression, resulting in atrophy in myotubes and muscle fibers [57]. The expression of FOXO transcription factors has been reported to be increased in numerous mouse models of cancer cachexia, with the attenuation of its expression leading to a decrease in muscle wasting. The use of targeted RNA oligonucleotides to suppress FOXO1 leads to an augmentation of skeletal muscle mass in mice affected by cachexia [58].

Atrophying myotubes lack the transcription factor JunB, an activator protein-1 (AP-1) family member, in the nucleus. The overexpression of JunB leads to muscular hypertrophy that is not influenced by the AKT/mTOR pathway. The transfection of JunB into muscles that have undergone denervation inhibits the interaction between FOXO3 and promoters of MURF1 and Atrogin-1, resulting in a decrease in muscle protein degradation [59]. In a manner akin to IGF-1, the signaling pathway including stromal cell-derived factor 1 (SDF1) and its receptor CXCR4, can exert a positive regulatory influence on the process of skeletal muscle development [60]. The expression levels of some genes belonging to the CXCR4 family, including SDF1 and p21-activated kinase 1 (PAK1), are decreased in skeletal muscles undergoing atrophy. The expression of SDF1 and CXCR4 in the skeletal muscle of individuals with cancer exhibits an inverse correlation with the expression of MURF1 and Atrogin-1. The upregulation of SDF1 or CXCR4 has been observed to mitigate muscle atrophy and enhance muscle fiber diameter, suggesting that the activation of the CXCR4 pathway could potentially counteract muscle wasting observed in cancer [61].

Overall, the signaling of IGF-1 is more prevalent than that of MSTN, whereas an excessive production of MSTN inhibits IGF-1 [62,63]. Of note, the administration of IGF-1 therapy has the capacity to counteract the inhibitory effects of myostatin on Akt. These data suggest that the pharmacological application of IGF-1 could be advantageous, even in situations in which myostatin or Activin are present [62]. Nevertheless, the association between IGF1 and cancer growth has raised several concerns, while several other mediators have been identified as key mediators of the cachexia process (i.e., Atrogin1 (MAFbx) and MuRF1 (Trim63)) [9]. Furthermore, the overexpression of genes such as MyoD and myogenin has been associated with muscle differentiation [9,64].

Another mechanism that may cause cancer-induced muscle wasting is primarily regulated by the TGF-β target gene known as KLF10 [65]. The Kruppel-like factor (KLF) family plays a pivotal role in various essential processes related to the development, maintenance, and metabolic regulation of skeletal muscle [66]. The overexpression of KLF10, a member of the KLF family, may be involved in the pathogenesis of cachexia relative to the induction of TGF-β [67,68]. Hence, the loss-of-function of KLF10 has been identified as a promising approach to mitigate cancer-induced muscle atrophy [69]. Furthermore, the activation of the TNF-α/TAK-1 signaling pathway leads to a rise in Activin A levels in skeletal muscle, in addition to myostatin [70]. This pathway indicates the potential involvement of TNF-α in the development of cachexia. TNF-α, a cytokine known for its proinflammatory properties, is widely regarded as an important component in cancer progression [71]. The role of TNF-α has been attributed to its involvement in crucial cellular processes such as cell proliferation, apoptosis inhibition, angiogenesis promotion, and the development of metastasis. TNF-α has inhibitory effects on the maturation of adipocytes and skeletal myocytes, as well as the development of insulin resistance through the disruption of the insulin signaling pathway [72,73,74]. The pathway of NF-κB, which is activated by the TNF-α/TAK-1 signaling, may play an important role in cachexia. Specifically, the activation of transforming growth factor-β-activated kinase 1 (TAK1) serves as a crucial activator of cellular apoptosis. Various internal and external stimuli, including microbial lipopolysaccharide, interleukin-1 (IL-1), and TNF-α, can trigger TAK1. In addition to its known antiapoptotic function, TAK1 is a mediator of necroptosis and an initiator for cell signaling, resulting in the activation of NF-κB as well as p38 MAP kinase [75]. In turn, these mediators induce E3 ligase genes by TNF-α, leading to the degradation of myofibrillar proteins through the ubiquitin-proteasome pathway and inhibition of protein synthesis (Figure 2). It is plausible that tumors or host tissues may secrete many cachectic factors, and therefore, a focus on exclusively one factor is insufficient [9].

Interleukin-6 (IL-6) has also been identified as a potential factor that interacts with TNF-α or acts independently to induce systemic inflammation in the context of cancer cachexia [76,77]. Multiple types of cancer have been associated with this particular interleukin, and proinflammatory cytokines that originate from the host, such as IL-1, have the potential to amplify its effects [9]. The association between IL-6 and bone morphogenetic protein (BMP) signaling has been described in the context of cachexia [78]. The regulation of muscle homeostasis has been demonstrated to be influenced by BMP signaling through the SMAD1/5/8 pathway [79]. The E3 ubiquitin ligase Fbxo30, also known as Musa1 (Muscle Ubiquitin ligase of the Skp, Cullin, F-box–containing (SCF) complex in Atrogin-1), plays a crucial role in the development of neurogenic muscle atrophy. Its regulation is negatively influenced by the BMP-SMAD1/5/8 axis [65].

In the context of catabolic conditions such as prolonged fasting or denervation, the inhibition of BMP signaling through experimental means leads to the suppression of Fbxo30 (Musa1) transcription that subsequently results in an excessive loss of muscle mass and weakness, which closely resembles the phenotype observed in cachexia [51,79]. The Noggin protein might also play an important role in cachexia. The Noggin protein mitigates the impact of BMPs on both muscle fibers and motor neurons [78]. The expression of the BMP inhibitor Noggin in muscle due to IL-6 leads to several outcomes, including neuromuscular junction (NMJ) disruption, denervation, and muscle atrophy [40]. Despite several roles of IL-6 in the context of cachexia, the exclusive targeting of IL-6 may encounter similar obstacles as anti-tumor necrosis factor (anti-TNF) therapy [9].

Another mechanism proposed for muscle atrophy is the reduction in the ribosomal capacity of the muscle in the setting of cancer [80,81]. Preclinical models of cancer have repeatedly demonstrated a decrease in skeletal muscle anabolism and protein synthesis [82,83]. These effects are primarily influenced by the amount of muscle ribosomes, which is controlled by the transcription of ribosomal (r)RNA genes (rDNA). An altered process of rDNA transcription has been suggested as one of the underlying causes for the decreased ribosomal capacity observed during cachexia. This impairment results in a significant reduction in the levels of rRNA [84].

Additionally, a recent study noted that apart from a decrease in rDNA transcription, the degradation of rRNA can also contribute to the deficiency of ribosomes and muscle atrophy in cancer models [83]. An increase in NUFIP1 mRNA levels, which acts as a receptor for the specific transportation of ribosomes to autophagic vesicles during ribophagy was previously reported. NUFIP1 is a protein that shuttles between the nucleus and cytoplasm attaches to ribosomes and guides them to lysosomes, where the NUFIP1-ribosome complexes are broken down [85]. The rise in NUFIP1 mRNA and the decreasing amount of NUFIP1 protein, along with an elevation in the ratio of LC3II (microtubule-associated protein light chain 3 which localizes to the autophagosomes) to LC3I (cytosolic form of LC3), suggested that ribophagy actively breaks down ribosomes during muscle wasting [86]. A better understanding of how the muscle’s ribosomal capacity is regulated can help explore novel methods to prevent muscle wasting in cancer cachexia.

In summary, muscle mass atrophy is a multifaceted phenomenon in cancer cachexia, orchestrated by a complex network of interdependent factors. Systemic inflammation, driven by cytokines such as TNF-a, IL-6 and IFN-γ, plays an important role in the initiation and progression of muscle wasting. These inflammatory mediators not only directly induce proteolysis, but also disrupt the anabolic-catabolic balance, skewing muscle homeostasis toward catabolism [87]. At the same time, metabolic derangements characterized by hypermetabolism, and lipid mobilization further exacerbate the muscular deprivation of essential nutrients needed to ensure homeostasis. The tumor itself further secretes specific factors that can accelerate muscle protein degradation [88]. The ubiquitin-proteasome pathway along with autophagy-lysosome systems are also upregulated in cachexia, further accelerating protein turnover and muscle atrophy. The combination of all these processes results in a relentless decline in muscle mass and strength which characterizes cachexia. A nuanced understanding of all different pathways is imperative for the development of targeted therapeutic strategies.

### 4.2. Fat Atrophy 

**Figure 3:**
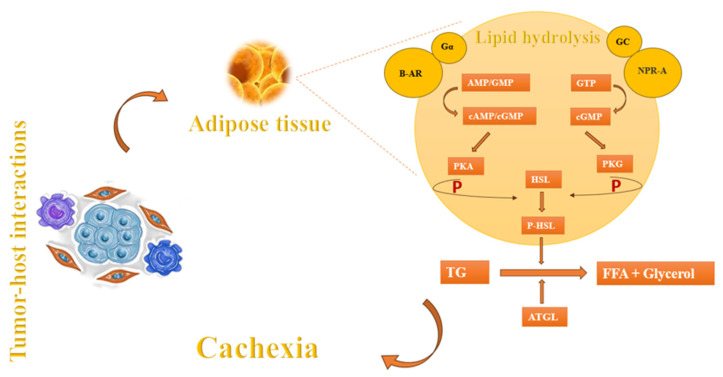
Cachexia-induced fat atrophy regulation mechanisms.

In addition to muscle wasting, another component of cachexia is fat atrophy [9]. Apart from factors such as anorexia and nutritional problems that arise during cancer progression, the main causes of fat atrophy may include lipolysis, diminished lipid uptake, and decreased de novo synthesis [4]. The process of lipolysis begins with the presence of lipid-mobilizing factors that initiate the breakdown of fat by interacting with β-adrenoreceptors (β-AR) and G protein-coupled receptors (Gα). These receptors play a crucial role in the production of cyclic adenosine monophosphate (cAMP) and cyclic guanosine monophosphate (cGMP). These cyclic nucleotides act as signals to activate protein kinase A (PKA), which subsequently phosphorylates hormone-sensitive lipase (HSL). HSL, in turn, facilitates the breakdown of triglycerides (TG) into free fatty acids (FFA) and glycerol, which are then released into the circulatory system [89]. Natriuretic peptide (NP) can also induce lipolysis by activating the NP receptor-A (NPR-A), which leads to an increase in guanylyl cyclase (GC)/cyclic-GMP (cGMP) and the activation of protein kinase G (PKG). PKG, possesses the capacity to attach a phosphate group to HSL which, in turn, proceeds the lipolysis. In addition, adipose triglyceride lipase (ATGL) plays a crucial role alongside HSL in the breakdown of adipose triglycerides (Figure 3). These mechanisms are also observed in the setting of cancer leading to cachexia [4,9,89].

In the setting of cancer cachexia, there is a significant reduction in the activity of lipoprotein lipase (LPL) in white adipose tissue (WAT). LPL is the enzyme responsible for breaking down both endogenous and exogenous triacylglycerols—located in lipoproteins—into glycerol and fatty acids. This decrease in LPL activity hinders the uptake of lipids, as the enzyme is essential to facilitate the entry of fatty acids into WAT [89]. Moreover, there is a reduction in de novo lipogenesis in adipose tissue in both mice and humans with tumors. This reduction leads to a drop in esterification, as the supply of fatty acids to synthesize triacylglycerols is diminished. Consequently, there is a decrease in the deposition of lipids, particularly triacylglycerols [89].

Brown adipose tissue (BAT) may also be affected by cancer cachexia. Previous studies have examined the correlation between tumor-derived IL-6 and β3-AR activation, as well as cancer cachexia-induced adipose tissue browning in mice genetically engineered to develop cancer cachexia [90,91,92]. Neutralization of IL-6 or β3-AR has a substantial positive impact on alleviating cancer cachexia [93]. The potential involvement of BAT activation in cancer cachexia remains uncertain, however. Nevertheless, tumor cell-derived IL-6 and PTHrP may have a significant impact on cancer cachexia through the activation of BAT and/or adipose tissue browning, particularly in animal tumor models [90]. 

### 4.3. Non-Coding RNAs

The term “non-coding RNAs” (ncRNAs) refers to a collection of transcripts that have limited or no capacity to be translated to proteins. These RNA molecules still play significant roles in the initiation and maintenance of cachexia while functioning as potential biomarkers [94]. ncRNAs exhibit variations in their length, encompassing both short forms, such as microRNAs (miRNAs), small interfering RNAs (siRNAs), small nuclear RNAs (snRNAs), piwi-interacting RNAs (piRNAs), as well as long forms, known as long ncRNAs (lncRNAs) [95]. miRNAs, a class of non-coding RNAs, have been a topic of increased focus due to their substantial role in gene expression regulation [96]. MiRNAs exert control over gene expression by binding to corresponding sequences on target messenger RNAs (mRNAs), leading to a reduction in mRNA levels [97]. Various illnesses and inflammatory processes are correlated with distinct miRNA expression profiles [98]. MiRNAs have the ability to affect the expression of target genes either through direct interactions or indirectly by modulating the activity of transcription factors, which subsequently regulate gene expression [99].

miRNAs have a significant impact on lipid metabolism, glucose homeostasis, and the development of metabolic tissues such as the liver, muscle, and adipose tissue [100]. Several miRNAs, including miRNA-378, miR-122-5p, miR-27b-3p, miR-375, and miR-424-5p, have exhibited alterations that are associated with the severity of cancer cachexia [101]. These alterations may contribute to the development of the observed wasting syndrome by activating pathways that lead to muscle wasting and the breakdown of adipose tissue [8,102]. Consequently, these miRNAs represent potential targets for pharmacological intervention in the treatment of cachexia [103,104]. 

### 4.4. JNK Signaling Pathway

Jun N-terminal kinase (JNK) signaling is an alternative pathway related to the development of cancer cachexia. JNK, often referred to as stress-activated protein kinase (SAPK), is one of the three primary constituents of the mitogen-activated protein kinase (MAPK) superfamily, alongside extracellular signal-regulated kinase (ERK) and p38 MAP kinase [105]. Muscle atrophy is characterized by a significant pattern of increased proteolysis resulting from enhanced autophagy and the activation of ubiquitin ligases that facilitate muscle protein degradation through the ubiquitin proteasomal pathway [106]. The E3 ubiquitin ligases Atrogin1 (MAFbx) and MuRF1 (Trim63) are key factors involved in the development of cachexia, with a prominent role in promoting proteolysis [107,108]. In addition, p38 and extracellular signal-regulated kinase (ERK) mitogen-activated protein kinases (MAPKs) promote protein breakdown through the stimulation of Trim63 and Fbxo32 gene expression [109,110]. The JNK signaling pathway has a direct role in regulating skeletal muscle protein turnover among patients with pancreatic cancer cachexia [111,112]. Both in vitro and in vivo models of cancer cachexia have demonstrated activation of JNK, along with its upstream activators and downstream targets. This pathway is also important in terms of cancer cachexia pathophysiology, as blocking JNK can prevent differentiated C2C12 myotubes from thinning caused by conditioned media (CM) [113]. The inhibition of TLR7/8/9, which are receptors involved in immune response, can also lead to a reduction in phosphorylated JNK levels. This inhibition can mitigate cancer cachexia through the prevention of microRNA-induced JNK-dependent cell death [105,112].

### 4.5. SIRT1–NOX4 Signaling

The proteins belonging to the silent information regulator 2 (SIR2) family, known as sirtuins, predominantly function as NAD^+^-dependent protein deacetylases and mono-[ADP-ribosyl] transferases. Sirtuins play a crucial role in linking alterations in energy metabolism with transcriptional reprogramming [114,115]. SIRT1, a member of the sirtuin family, can influence muscle physiology through the modulation of muscle cell differentiation and proliferation [116]. This protein plays a significant role in regulating the creation of reactive oxygen species (ROS); the depletion of SIRT1 in cancer cells boosts NF-B signaling in cachectic muscles, leading to increased expression of FOXO transcription factors and NADPH oxidase 4 (Nox4), a critical regulator of ROS production [38,117]. A recent study demonstrated a negative correlation between the expression of NOX4 and the cross-sectional area of skeletal muscle fibers among individuals diagnosed with pancreatic cancer [118]. In turn, the signaling axis, including SIRT1 and NOX4, may be a regulator of cellular senescence and oxidative stress, which may act as a cachexia regulator [118,119].

### 4.6. Toll-like Receptor/MyD88/XBP1 Signaling Axis

The endoplasmic reticulum (ER) plays a significant role in the synthesis, folding, and maturation of cellular proteins within mammalian cells. The ER also serves a crucial function in the cellular regulation of calcium levels [120]. Misfolded or unfolded proteins, as well as disturbances in calcium homeostasis, can induce ER stress [121]. The resolution of stress is achieved through the activation of a signaling pathway known as the unfolded protein response (UPR) [122]. Levels of markers of ER stress and UPR pathways appear to be increased in skeletal muscle under a range of catabolic conditions, including cancer [123]. Bohnert et al. demonstrated that the Lewis lung carcinoma (LLC) model of cancer cachexia resulted in an upregulation of Toll-like receptors (TLRs) and myeloid differentiation primary response gene 88 (MyD88) expression in skeletal muscle [123,124,125]. The regulation of downstream signaling pathways involved in the activation of the interleukin-1 (IL-1) receptor and Toll-like receptor (TLR) is controlled by the indispensable adaptor protein known as MyD88 [126]. This gene expression is essential for regulating skeletal muscle growth in a number of circumstances [124,125]. For example, MyD88 facilitates the fusing of myoblasts in a cell-autonomous manner, both during postnatal muscle growth and overload-induced hypertrophy [127]. In contrast, the activation of TLRs and the MyD88 signaling pathway stimulates an inflammatory response, thereby exacerbating the muscular pathology observed in animal models of muscular dystrophy [128].

The activation of the unfolded protein response (UPR) in skeletal muscle during cancer cachexia is facilitated by TLR/MyD88; in turn, the targeted removal of MyD88 reduces the muscle atrophy induced by LLC tumors in mice [127,129]. In a manner akin to MyD88, the targeted elimination of XBP1, specifically in skeletal muscle, mitigates the detrimental impact of LLC tumor-induced muscle wasting, both in vivo and in vitro [123,130,131]. Moreover, the activation of sXBP1 effectively induces atrophy and enhances the gene expression of specific components of the ubiquitin-proteasome system (UPS), proinflammatory cytokines, and autophagy in cultured myotubes [123,132]. Further investigation is needed to characterize the involvement of MyD88, as well as elucidate the specific pathways that modulate skeletal muscle mass in cancer-induced cachexia.

### 4.7. TRIF

MyD88 serves as a significant inflammatory signaling pathway in the context of illness and cancer. Mice lacking the MyD88 gene (referred to as MyD88 knockout or MyD88KO mice) exhibit sickness behavior following exposure to inflammatory stimuli [125,133]. Following the administration of systemic lipopolysaccharide (LPS) challenge, MyD88KO mice exhibit an increased expression of various cytokine and chemokine genes inside the hypothalamus [125,133]. The adapter protein known as TIR-domain-containing adaptor-inducing interferon (TRIF) plays a significant role in modulating inflammatory signaling. Hence, the assessment of the inflammatory signaling TRIF pathway both in the setting of acute inflammation (namely, the LPS challenge) and chronic inflammatory conditions (such as cancer cachexia) is warranted [125,133]. The impact of the TRIF pathway on central nervous system (CNS)-mediated changes in behavior and metabolism during illness has been relatively understudied [134]. Following systemic or central exposure to LPS, the activation of TRIF signaling plays a crucial role in the development of neuroinflammation and subsequent acute sickness behavior [124,125]. Interestingly, mice with a deficiency in TRIF have a reduction in the occurrence of cancer cachexia [135,136]. These findings suggest that TRIF may be a potential therapeutic target for cachexia and an important inflammatory-driven signaling pathway related to metabolic changes during illness and cancer [125,137].

## 5. Potential Molecular Interventions

The lack of a widely accepted treatment for cancer cachexia remains a challenge, mostly due to the various cancer types and pathophysiological mechanisms involved in the cachexia process. Implementing strategies that target multiple pathways implicated in the development of cachexia can potentially mitigate this problem. As mentioned, the overexpression of MURF1 and Atrogin-1, which are crucial regulators in muscle atrophy, contributes to the development of cachexia. Consequently, these molecules hold promise as prospective targets for therapeutic interventions in the management of cachexia [45]. As MURF1 and Atrogin-1 directly control protein degradations that lead to cachexia, the inhibition of MURF1 and/or Atrogin-1 may preserve protein levels and maintain muscle mass without unwanted side effects. In addition, tumor cells have the ability to release cytokines such as TNF-α, IL-1, IL-6, etc. This secretion has the capacity to activate NF-κB, which in turn may contribute to the development of cachexia. The modulation of these cytokines by the JAK-STAT pathway may be a potential therapeutic target for the mitigation of cachexia [45,138,139,140,141]. 

Another field of study related to the treatment of cancer cachexia has focused on the TGF-β superfamily. Many cancer patients experiencing cachexia have elevated blood levels of myostatin and activin [142]. The development of antibodies capable of inhibiting the actions of both myostatin and activin could, therefore, serve as a potential approach to mitigate muscle atrophy [143,144,145]. The crucial carcinogenic pathway PI3K/AKT/mTOR—also responsible for protein biosynthesis—is also effectively suppressed by myostatin and activin [49,50]. In contrast, Insulin-like growth factor 1 (IGF-1) is an important activator of the PI3K/Akt signaling pathway, which plays a crucial role in protein synthesis and the promotion of muscle growth [45,146]. As such, the potential therapeutic application of IGF-1 in the treatment of cancer cachexia should also be considered. It is important to note, however, that there are certain side effects associated with IGF-1 administration [7,37], including toxicity resulting from elevated blood glucose levels due to the action of IGF-1, as well as the promotion of tumor growth induced by hormone involvement, which may restrict the effectiveness of IGF-1 in preventing cachexia [147,148].

## 6. Conclusions

Research primarily conducted in animal models has indicated that MURF1 and Atrogin-1, JAK-STAT pathway, TGF-β, Toll-like receptors (TLRs), c-Jun N-terminal kinases (JNK), sirtuin 1 (SIRT1)-NADPH oxidase 4 (NOX4) signaling pathways, and several miRNA species, represent potential therapeutic targets to reverse/treat cachexia. Nevertheless, a definitive treatment to reverse cancer cachexia remains elusive [149,150,151,152]. The adoption of multimodal therapies, including the integration of pharmacological interventions coupled with physical activity, holds the most promise to mitigate muscle atrophy while improving overall well-being and potentially extending the survival of individuals with cancer [78,102,118].

## Figures and Tables

**Table 1:** Summary of potential biomarkers.

Origin	Potential Biomarkers ^1^
Cachexia-inducing factors	ActA, MSTN, GDF15, PTHrP, ZAG, Ang II, MURF-1, Atrogin-1
Inflammatory factors	TNF-α, IL-6, IL-1β, IL-8, MCP-1, CRP, Albumin
Muscle and fat wasting products	β-dystroglycan, Glycerol, FFA, HCERs, LCERs
MicroRNAs	MicroRNA-21, MicroRNA-203, MicroRNA-130a
Other factors	CNDP1, TIMP-1

^1^ ActA (Activin A), MSTN (Myostatin), GDF15 (Growth/differentiation factor 15), PTHrP (Parathyroid Hormone release Peptide), ZAG (Zinc-α2-glycoprotein), Ang II (Angiotensin II), MURF-1 (muscle RING-finger protein-1), MCP-1 (Monocyte chemoattractant protein-1), CRP (C-reactive protein), FFA (Free fatty acid), HCERs (Hexosyl-ceramides), LCERs (Lactosyl-ceramides), CNDP1 (Carnosine dipeptidase 1), TIMP-1 (Tissue inhibitor of metalloproteinases-1).

## Data Availability

No new data were created.
